# Driving antibiotic stewardship awareness through the minibus-taxi community across the Tshwane District, South Africa—a baseline evaluation

**DOI:** 10.1093/jacamr/dlab106

**Published:** 2021-08-07

**Authors:** Tumelo T W Mokoena, Natalie Schellack, Adrian J Brink

**Affiliations:** 1 Division of Clinical Pharmacy, School of Pharmacy, Sefako Makgatho Health Sciences University, Molotlegi Street, Ga-Rankuwa, Gauteng, South Africa; 2 Department of Pharmacology, Faculty of Health Sciences, University of Pretoria, Pretoria, Gauteng, South Africa; 3 Division of Medical Microbiology, Faculty of Health Sciences, University of Cape Town and National Laboratory Sciences, Cape Town, South Africa

## Abstract

**Background:**

The minibus-taxi community plays an integral role within society, and for years this community has been neglected. Of late, studies on minibus-taxi operators’ health and their perceptions of HIV have emerged. Antibiotic resistance is a global problem and to help curb its spread studies have looked into the knowledge, attitude and perceptions amongst students and healthcare professionals, and yet little to nothing is known about the minibus-taxi community.

**Objectives:**

To assess the knowledge and understanding of the minibus-taxi community on antibiotics and antibiotic resistance, and document indigenous antibiotic terminology used across the Tshwane District in Gauteng, South Africa.

**Methods:**

A semi-structured questionnaire was adopted from WHO, translated into commonly spoken languages and administered to 83 minibus-taxi community members: 27 minibus-taxi operators and 56 commuters. A convenience sampling method was utilized in selecting the minibus-taxi ranks and routes. The questionnaire was later adapted to the minibus-taxi community’s busy lifestyle and a section added to document antibiotic terms.

**Results:**

Seventy-one percent (*n *=* *59) of the participants knew the importance of taking antibiotics as directed, while 64% (*n *=* *53) believed it’s correct to share antibiotics. Seventy-five percent (*n *=* *62) thought antibiotic resistance occurred in the human body. One misconception noted was that the minibus-taxi community thought antibiotics treated cold/flu and fever. Over 80% of the community were unfamiliar with antibiotic terminology.

**Conclusions:**

Several misconceptions were documented amongst the minibus-taxi community and, whilst highlighting the linguistic barriers for the term antibiotic resistance, we identified several enablers for public awareness and empowerment. Further studies are required to define appropriate indigenous terms for future educational antibiotic campaigns.

## Introduction

The terms ‘antibiotic resistance’, ‘antimicrobial resistance (AMR)’ and ‘superbugs’ are not instantly understood by people, and this highlights the need to find simpler terminology.^1^ Patients often interpret ‘resistance’ as being part of the human body instead of the bacteria. Healthcare professionals use the term daily amongst themselves with the assumption that patients understand its meaning.^2^ In a number of surveys done worldwide, it was concluded people had a lack of knowledge and understanding of antibiotics.^1–5^

South Africa has a high disease burden, with HIV/AIDS, TB and sexually transmitted infections (STIs) being the most prevalent.^6^ MDR microorganisms have made HIV/AIDS, TB and STIs difficult and expensive to manage, causing healthcare professionals to resort to prolonged treatment durations in order treat patients.^7^ Poor adherence to the standard treatment guidelines (STG) coupled with the high patient volumes in primary healthcare facilities have further contributed to the development of antimicrobial resistance,^8^ forcing WHO to increase public health education campaigns and communication that target members of the public as a strategy to curb resistance.^9^

AMR has been viewed as an occurrence in the body,^2^ and it seems AMR and associated terms are meaningless to the public.^1^ This has drawn special attention towards the lack of awareness surrounding the language of AMR.^10^ Antimicrobial resistance campaigns have mostly been found ineffective in educating the public,^11^ and also revealed that these antimicrobial campaigns result in further confusion of the public.^12^

South Africa is diverse and comprises 11 official languages, which are IsiZulu, isiXhosa, Afrikaans, Sepedi, Setswana, English, Sesotho, Xitsonga, SiSwati, Tshivenda and isiNdebele in the order most spoken to least spoken.^13^^,^^14^ Information is well understood when conveyed and processed in a first language,^15^ and correct terminology allows better communication between healthcare professionals and laymen.

The minibus-taxi industry plays an integral part in society and is the most abundant form of transportation available to the public as they are affordable and used by the low-middle income group. The taxi industry also forms the backbone of the public transport industry and accounts for the bigger chunk of daily public commuting.^16^ Minibus-taxis make over 15 million commuter trips daily in South Africa, and from the various public transport choices available (train, bus and minibus taxis), the minibus taxis reign supreme at 67%, with trains lagging behind at 13% being the least chosen form of public transport for many South Africans.^17^

The minibus taxi is typically a 16-seater minibus that operates along fixed routes; they carry commuters and stop anywhere for commuters. The commuter hails the minibus taxi using hand signals as an indication that they are travelling to a certain destination. Fares are paid in cash and may differ from area to area. Fares are set by the taxi association or council and are based on petrol price, consumer availability and minibus operator. A minibus-taxi trip to work averages 50 min with an additional average transit time of 15 min on foot to get to a taxi rank. Taxis depart and converge at a central point known as a taxi rank where there are flea markets.^16^^,^^17^

The taxi industry is predominantly male, with operators who are aged between 25 and 44 years.^18^ The taxi operators receive their remuneration on a quarterly basis or as a fixed amount per day based on daily targets that are agreed upon between taxi operator and taxi owner.^19^ These daily targets have resulted in long working hours, causing increased stress levels, unhealthy diet, lack of exercise, smoking and consumption of alcohol and stimulants, which all have contributed to operators having various chronic and acute illnesses.^19^ This has resulted in the taxi industry being the subject of several studies such as assessments of health^19^ and infectious diseases burden.^20^ Other studies have explored the attitudes and perceptions of taxi operators of HIV and AIDS.^18^^,^^21^

Targeting the taxi operators and taxi commuters, also collectively referred to as the taxi community, for public awareness stewardship campaigns might represent an important enabler to effect changes in antibiotic utilization in South Africa. This pilot study therefore seeks to describe the baseline understanding of the knowledge, attitude and perceptions of the taxi community of antibiotics and antibiotic resistance, with the aim to document the use of indigenous antibiotic terminology.

## Methods

### Setting

The City of Tshwane District is situated in Gauteng, South Africa. The Tshwane District has a commuter population of 1 022 490 and in 2018 the taxi business reported 6900 minibus taxis in operation around the Tshwane District.^16^

### Participant recruitment and data recording

The study questionnaire was adjusted and translated into IsiZulu and Setswana which were mostly spoken by the taxi community. Taxi ranks were selected as part of a convenience sampling where the questionnaires were handed to participants. The questionnaire was piloted initially on 15 participants from different taxi ranks around the City of Tshwane District in Gauteng.

Taxi commuters were approached at different malls/shopping centres near taxi ranks. The aims and objectives of the study were fully explained to them prior to the commencement of filling the questionnaire. The participants signed consent forms. Taxi operators were approached in their taxis while resting, sitting in groups having conversation. The most convenient time to approach the taxis was between 9 am and 12 pm.^18^ The questionnaire was self-administered, or researcher-administered should the participant request to be assisted. The study was quantitative in nature using a semi-structured questionnaire derived from WHO (the questionnaire is available as Supplementary data at *JAC-AMR* Online).^22^ The questionnaire consisted of four sections, and the details in each section are summarized in Table 1.

**Table 1.** Summary of the questionnaire sections

Section	Summary
i	This section captured the demographics and level of education of the taxi community.
ii	This section focused on knowledge and understanding of antibiotics and consisted of four statements that tested the general understanding of antibiotics (conditions treated with antibiotics—the conditions ranged from major illnesses such as HIV/AIDS to minor headaches, some conditions were treatable by antibiotics whilst others weren’t—and when to stop antibiotic treatment) that could be answered by simply selecting an answer.
iii	This section tested the awareness of commonly used antimicrobial terms, knowledge and understanding of antibiotic resistance and the source of these terms. This section of the study focused on the participants’ familiarity with terminology around antibiotics; the terms in question were: ‘antibiotic resistance’, ‘superbugs’, ‘antimicrobial resistance’, ‘AMR’, ‘drug resistance’, ‘antibiotic-resistant bacteria’ and ‘antibiotic stewardship’.
	The National Department of Health (NDoH) recommended that healthcare professionals educate patients on antibiotic resistance (NDoH, 2014); this study further aimed to explore which of the different forms of information sources—such as doctors, nurses, pharmacists, family members or friends, social media, media or specific campaigns—were most effective at educating the public. The taxi commuters’ understanding of antibiotics were further tested on seeing how they responded to statements on antibiotic resistance interventions. The Likert scale was used ranging from 1 to 5 (1 = disagree strongly, 2 = disagree slightly, 3 = neither agree nor disagree, 4 = agree slightly, 5 = agree strongly).
iv	This section aimed to document terminology used by the taxi community around the Tshwane District. The participants were asked to translate the following terms: ‘antibiotic/s’, ‘antibiotic resistance’, ‘antibiotic stewardship’, ‘infection’, ‘infection prevention’, ‘bacteria’, ‘microorganisms’ and ‘superbugs’ into their various indigenous languages. This was to establish if these terms existed and if they were meaningful to society. The calculated population size for this study was 748; only 83 participants were interviewed.

The checklist was subsequently used to capture the following information, as outlined by the objectives set in the four sections: demographics; knowledge and understanding of antibiotics; awareness and sources of antimicrobial terms; and antimicrobial terms in indigenous South African languages.

The data collected were imported to a Microsoft Excel™ spreadsheet by the researcher; a second data person was asked to import the data to a separate Microsoft Excel™ spreadsheet and the results were compared to ensure validity reliability and dependability of the data.

### Ethics

Ethical approval was obtained from the Sefako Makgatho Health Sciences University Research Ethics Committee (SMUREC) with the ethics reference number: SMUREC/P/57/2019: PG. No participants’ names, cell phone numbers and physical addresses were obtained during the data collection process and confidentiality was maintained at all times. Participants were identified using unique identity numbers allocated to them by the researcher.

None of the participants in the study received incentives; the researcher approached participants individually or in groups to briefly explain the purpose of the study. Interested parties would then ask for the questionnaire, which took 5–10 min to fill out. Some of the taxi operators required assistance in answering the questionnaire.

## Results

A total of 83 taxi community members completed the questionnaire between the months of July and August 2019. Of the 83 participants, 67% (*n *=* *56) were minibus-taxi commuters and the remainder 33% (*n *=* *27) were minibus-taxi operators.

### Demographics

More than half of the participants in this study were male (60%; *n *=* *50). Ten of the eleven official languages were recorded, and 22% (*n *=* *18), 19% (*n *=* *16) and 17% (*n *=* *14) of the participants spoke Sepedi, isiZulu and Setswana, respectively (Figure S1).

The age of the participants ranged between 18 and 64 years whilst the majority of participants were between the ages of 25 and 34 years (41%; *n *=* *34). The median age of the participants was 34 years (IQR 29–62 years).

The level of education of the minibus-taxi community was captured as follows: more than half of the participants (51.8%; *n *=* *43) were educated to 12th grade or less, whilst 37% (*n *=* *31) had a college education. Five (6%) of the participants held a bachelor’s degree and 2.4% (*n *=* *2) had a vocational certificate in their possession; only 2.4% (*n *=* *2) of participants had not completed schooling.

### Knowledge and understanding of antibiotics

#### ‘When do you think you should stop taking antibiotics once you’ve begun treatment?’

Nearly three-quarters (71%; *n *=* *59) of the participants took antibiotics as directed, followed by 27% (*n *=* *22) of participants who chose to stop antibiotics once they felt better. Only two of the participants didn’t know when to stop antibiotics.

#### ‘Is it okay to use antibiotics that were given to a friend or family member, as long as they were used to treat the same illness?’

Sixty-four percent (*n *=* *53) of participants chose true, and 8% (*n *=* *7) didn’t know, whilst 28% (*n *=* *23) said they would not share their antibiotics.

#### ‘Which of these conditions can be treated with antibiotics?’

Figure S2 provides a summary of the percentage of participants who either agreed or disagreed on conditions treatable with antibiotics. Sixty-seven percent (*n *=* *56) of the participants agreed that antibiotics can treat a cold and flu, whereas 59% (*n *=* *49) believed antibiotics can treat a fever, and 51% (*n *=* *42) selected that antibiotics can treat a sore throat.

### Knowledge and understanding of antibiotic resistance

When participants were asked about their knowledge of antibiotic terminology, over 80% of the participants had never heard of the following terms: ‘antibiotic stewardship’, ‘antibiotic-resistant bacteria’, ‘drug resistance’, ‘AMR’ and ‘antimicrobial resistance’, whereas 51% (*n *=* *42) and 52% (*n *=* *43) had heard of the terms ‘antibiotic resistance’ and ‘superbugs’, respectively, from various sources including doctors, nurses and pharmacists (Figure S3).

**Figure 1.**
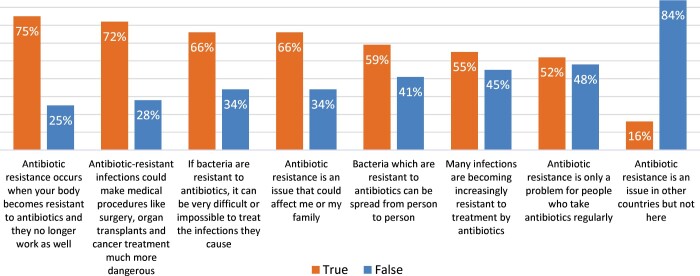
Taxi community’s understanding of antibiotic resistance statements.

Figure 1 depicts the taxi community’s understanding of antibiotic resistance statements. Seventy-five percent (*n *=* *62) of the participants stated that ‘antibiotic resistance occurs when your body becomes resistant to antibiotics’.

#### Commuters’ awareness of their role and that of others in addressing antibiotic resistance

**Figure 2.**
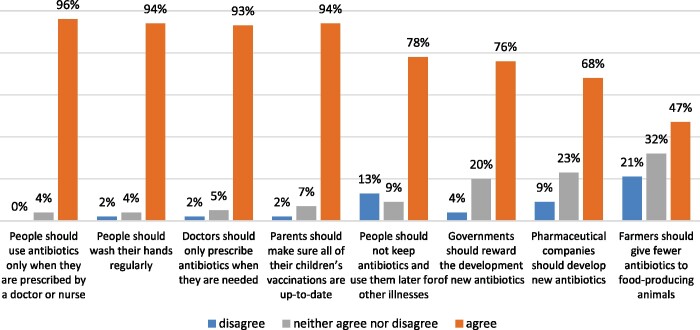
Participants’ level of awareness of ways to address antibiotic resistance.

The participants’ level of awareness of ways to address antibiotic resistance is depicted graphically in Figure 2.

Over 90% of participants agreed with statements on hand hygiene, finishing antibiotics as prescribed, doctors prescribing antibiotics only when necessary and keeping children’s vaccinations up to date as factors important to preserve antibiotics.

Ninety-one percent (*n *=* *51) of commuters agreed that using antibiotics properly is everyone’s responsibility, while 61% (*n *=* *34) agreed that medical experts will solve the antibiotic resistance crisis and 57% (*n *=* *32) agreed that there is not much they could do to halt antibiotic resistance.

### Indigenous languages—common antibiotic terms

Participants were asked to give their indigenous version of the following antibiotic terms: ‘antibiotics’, ‘antibiotic resistance‘, ‘antibiotic stewardship’, ‘infection’, ‘infection prevention’, ‘bacteria’, ‘microorganisms’ and ‘superbugs’ (Table S1). Over 55% (*n *=* *46) of participants responded with either, ‘I don’t know’, ‘I have never heard of such’, ‘I have never thought of it’ or ‘not applicable’. Eleven of the 12 official languages were recorded, and only 45% (*n *=* *37) of participants managed to provide terms; not all the terms could be documented as these terms were new to the participants.

## Discussion

Our pilot study amongst the minibus-taxi community identified several potential enablers to effect changes in antibiotic utilization in primary care in South Africa. The study demonstrated that participants have some level of understanding of antibiotics and AMR as a large proportion of the participants knew to take antibiotics as directed. This correlates with the recent findings of a South African patient study, where 90% felt it is important to complete a course of antibiotics as prescribed.^23^ However, sharing of antibiotics was a common practice for the taxi community and they shared based on the fact of like illnesses.

Furthermore, there is a misconception amongst the commuting participants with regards to the use of antibiotics for cold/flu and all fevers. This is the opposite to the findings of Bulabula *et al.*,^24^ where over 63% of pregnant women in Cape Town, South Africa would not use antibiotics to treat flu. In contrast, just over half of our participants would not use antibiotics to treat HIV/AIDS. This may be the consequence of South Africa’s high disease burden of HIV/AIDS and TB,^6^ where health campaigns have mainly focused on these diseases to improve people’s knowledge and understanding of HIV/AIDS and how it is treated.^25^ This suggests that targeted public awareness campaigns may be effective in changing antibiotic perceptions and attitudes.

Provision of antibiotic keywords along with a list of knowledge sources identified several other potential interventions. Just under 50% of participants had not heard of the terms ‘antibiotic resistance’ and ‘superbugs’, despite doctors, nurses and pharmacists playing a role in educating our study participants. In contrast, the majority of the participants had never heard of the keywords, ‘antimicrobial resistance’, ‘AMR’, ‘drug resistance’, ‘antibiotic-resistant bacteria’ and ‘antibiotic stewardship’.

Participants were aware that AMR is driven by incorrect use of antibiotics, but they felt they had no responsibility in preventing its spread. The public can play a decisive role in antimicrobial stewardship and more local directives are required in the education of patients by healthcare professionals at the point of care.^26^ Despite the participants’ perceived understanding of antibiotics and AMR, AMR is conflictingly viewed as being part of the body and not bacteria, a similar trend documented in other studies.^2^^,^^23^ The perception relates to immunity and not being able to resist infections.

Not much is known on the understanding of antibiotic terminology in South African languages, and yet only the term ‘antibiotic’ was translated into other languages by the department of Arts and Culture in an effort to bridge the language gap between healthcare professionals and the public.^27^ Only half of the participants completed the questionnaire on indigenous antibiotic terminology as our urban participants had either heard these terms at some point but couldn’t put them in context or they never knew such terminology existed in their home language. Whilst the study documented 11 languages, variation of the same term was noted within a language. For example, in isiZulu the word ‘antibiotics’ is referred to as ‘amapilisi’ and ‘umjovo’ which translate into ‘pills’ and ‘syringes’, respectively. The same phenomenon occurred in Sepedi where the word ‘antibiotics’ is referred to as ‘dipilisi’ or ‘dihlare’ which translate to ‘pills’ and ‘medicine’, respectively. This suggests the urgent need to standardize a common reference for antibiotics and resistance across linguistic barriers nationally.

### Conclusions

Healthcare professionals play an important role in educating the public on their role in antibiotic stewardship, but the information is not necessarily translating into improved knowledge and attitudes. As a consequence, the minibus-taxi community studied do not have an appropriate level of understanding of antibiotics and AMR. Furthermore, antibiotic and resistance terminology would need to be defined and simplified for laymen to better understand and for use in bespoke awareness campaigns to mitigate rising AMR rates. The fundamental problem is a lack of official antibiotic terms in all South African languages. Interventions would also need to focus on defining and differentiating viruses and bacteria, along with their associated diseases, as a great deal of misconception was evident. A multifaceted strategy where educational interventions occur on many levels will have to be applied to our communities after addressing local barriers to change. These may be the only interventions with effect sizes of sufficient magnitude to potentially reduce the incidence of antibiotic-resistant bacteria.

## Funding

The study was funded by the Division of Clinical Pharmacy, School of Pharmacy, Sefako Makgatho Health Sciences University (SMU).

## Transparency declarations

None to declare.

## Supplementary data

Supplementary data (Questionnaire, Figures S1 to S3 and Table S1) are available as Supplementary data at *JAC-AMR* Online.

## Supplementary Material

dlab106_Supplementary_Data
