# Supplementary material for: Driving antibiotic stewardship awareness through the minibus-taxi community across the Tshwane District, South Africa—a baseline evaluation
Source: JAC Antimicrob Resist. 2021 Aug 7;3(3):dlab106. doi: 10.1093/jacamr/dlab106 (PMC8346700; doi:10.1093/jacamr/dlab106)
Supplement: dlab106_Supplementary_Data [file dlab106_supplementary_data.docx]

**Supplementary data**

**Questionnaire**

Driving antimicrobial stewardship and awareness through the taxi community across the Tshwane District, South Africa- a baseline evaluation.

**Gender**: Male / Female

**Age**: 18-24/ 25-34/ 35-44/ 45-54/ 55-64/ 65+

**Home language**: isiZulu/ isiXhosa/ Afrikaans/ Sepedi/ Setswana/ Sesotho/ Xitsonga/ SiSwati/ Tshivenda/ isiNdebele

**Level of education**: Primary school (grade 1-7) /High school (grade 8-12)/ no qualification/ Some college, diploma/ Technical/Vocational training or Associate degree/ Bachelor’s or Honors degree/ Master’s/ Doctorate degree

**SECTION II: KNOWLEDGE AND UNDERSTANDING - ANTIBIOTICS**

**1. When do you think you should stop taking antibiotics once you’ve begun treatment?**

a. When you feel better

b. When you’ve taken all of the antibiotics as directed

c. Don’t know

**2. Is it okay to use antibiotics that were given to a friend or family member, as long as they were used to treat the same illness**

a. True

b. False

c. Don’t know

**3. Which conditions can be treated with antibiotics? (Yes/ no/ I don’t know).**

a. HIV/AIDS

b. Gonorrhoea

c. Bladder infection or urinary tract infection (UTI)

d. Diarrhoea

e. Cold and flu

f. Fever

g. Malaria

h. Measles

i. Skin or wound infection

j. Sore throat

k. Body aches

l. Headaches

**SECTION III: KNOWLEDGE AND UNDERSTANDING - ANTIBIOTIC RESISTANCE**

**4a. Have you heard of any of the following terms? (Yes / No)**

a. Antibiotic Resistance

b. Superbugs

c. Antimicrobial Resistance

d. AMR

e. Drug Resistance

f. Antibiotic-resistant bacteria

g. Antibiotic Stewardship

**4b. where did you hear about these terms?**

If answered YES:

a. Doctor or nurse

b. Pharmacist

c. family member or friend (including on social media)

d. Media (newspaper, TV, radio)

e. Specific campaign

f. Other

**5. Please indicate whether you think the following statements are ‘true’ or ‘false’**

a. Antibiotic resistance occurs when your body becomes resistant to antibiotics and they no longer work as well

b. Many infections are becoming increasingly resistant to treatment by antibiotics

c. If bacteria are resistant to antibiotics, it can be very difficult or impossible to treat the infections they cause

d. Antibiotic resistance is an issue that could affect me or my family

e. Antibiotic resistance is an issue in other countries but not here

f. Antibiotic resistance is only a problem for people who take antibiotics regularly

g. Bacteria which are resistant to antibiotics can be spread from person to person

h. Antibiotic-resistant infections could make medical procedures like surgery, organ transplants and cancer treatment much more dangerous

**6. On the scale shown below, how much do you agree the following actions would help address the problem of antibiotic resistance?**

**(1-Agree Strongly 2-Agree Slightly 3-Neither agree nor disagree 4-Disagree Slightly 5-Disagree Strongly)**

a. People should use antibiotics only when they are prescribed by a doctor or nurse.

b. Farmers should give fewer antibiotics to food-producing animals.

c. People should not keep antibiotics and use them later for other illnesses.

d. Parents should make sure all of their children’s vaccinations are up-to-date

e. People should wash their hands regularly.

f. Doctors should only prescribe antibiotics when they are needed.

g. Governments should reward the development of new antibiotics.

h. Pharmaceutical companies should develop new antibiotics.

i. Antibiotic resistance is one of the biggest problems the world faces.

j. Medical experts will solve the problem of antibiotic resistance before it becomes too serious.

k. Everyone needs to take responsibility for using antibiotics responsibly.

l. There is not much people like me can do to stop antibiotic resistance.

m. I am worried about the impact that antibiotic resistance will have on my health, and that of my family.

n. I am not at risk of getting an antibiotic- resistant infection, as long as I take my antibiotics correctly.

**SECTION IV: TERMINOLOGY PERTAINING TO ANTIBIOTIC AND RESISTANCE**

**7. In your home language, what are the following terms?**

a. antibiotic/s:

b. antibiotic resistance:

c. antibiotic stewardship

d. infection

e. infection prevention

f. bacteria

g. microorganisms.

h. Superbugs

**Figure S1.** Percentage of participants by home language

**Figure S2.** Conditions treated with antibiotics according to the participants.

*urinary tract infection

**Figure S3.** Antibiotic terminologies and their sources

**Table S1.** African terminology for antibiotics

|  | **Antibiotics** | **Antibiotic resistance** | **Antibiotic stewardship** | **Infection** | **Infection prevention** | **Bacteria** | **Microorganisms** | **Superbugs** |
| --- | --- | --- | --- | --- | --- | --- | --- | --- |
| **IsiZulu** | Amapilisi |  |  | uguli |  | Amagerms | amagerms | mbungulu |
|  | Amacqiwane | Cwecwa | Umkhambathi | Ukuqula | Imvikelo yukugula | ukuncola |  | ukuncola |
|  | Amaphilisi |  |  | iciwane | amaphilisi wokuvikela iciwane | emajemisi |  |  |
|  | Umjovo | Ukungasebenzi komjovo ongamaphilisi | ukukundisa ngomjovo wama philisi | Amagciwane | ukuvikela amagciwane | Amagciwane | Amagciwane | Amagciwane amakhulu |
|  | Amaphilisi |  |  | Isifo |  | Igciwane |  | Imbungulu |
|  | Amaphilisi |  |  | Imikuhlane |  |  |  |  |
| **Sepedi** | Depilisi |  |  |  |  |  |  |  |
|  | Thibela bolwetsi |  |  | Phetelo | Thibela petelo | kokwana thloko |  |  |
|  | Philisi |  |  | Bolwetsi |  | Kokwana thloko |  |  |
|  | Dihlare |  |  | Pheteletso | go thibela malwetsi |  |  |  |
|  | Di ukubatsi |  |  |  | Thibela bolwetsi |  |  |  |
|  |  |  |  | Bolwetsi | Thibela malwetsi |  |  |  |
|  | Dihlare |  |  | go fetelwa | go thibela go fetelwa | ditshila |  |  |
|  |  |  |  | Fitelello | Ditlamorago | Twatsi | Tlakatlakano ya di lelo |  |
|  |  |  |  | Kokwane | tibela malwetsi |  |  |  |
|  |  |  |  | Tshwaetso ya malwetsi | Thibela malwetsi | Mogare wa bolwetse |  |  |
| **Sesotho** | Dipilisi |  |  | bolwetsi |  | dichila |  |  |
|  |  |  |  | Tshwaetso | Thibela tshwaetso | kokwana |  |  |
|  | Dipilisi |  |  |  |  | kokwana thloko |  |  |
|  |  |  |  | Tshiwahetsano | Ho thibela tshwanetsano | Kokwana hloko |  |  |
|  | Anti-biotics |  |  | Infection |  | Bacteria |  |  |
| i**siNdebele** | Mshoga | ukungabheregi kwe shlahla |  | Indruga | Isivhikhela bolwlao | ndonga | Bolwelwe |  |
|  | kwemithi elwa nama gciwane | ukumelana namagciwane | ubuphathi bama antibiotic |  | Ukuvikela ukutheleleka | amagciwane | Silwanyana |  |
| **isiXhosa** | Amapilisi wokhugeza igazi |  |  |  |  |  |  |  |
| **SiSwati** | Ukumelana nemithi elwa namagciwane | Ukwangamela ama-antibiotic | Ukutheleleka | ukuvimbela ukutheleleka | amagciwane | ama micro organisms | ama-superbug | Ukumelana nemithi elwa namagciwane |

**Table S1.** African terminology for antibiotics (continued)

| **Tshivenda** | Mushonga wa fhugudza nyaluwo ya zwitshili | U kona ha zwitshiliulwa na mushonga wa u zwi vhulaha | Ndi ndila ya ufunza vhafhi vha mushonga wa u zwi vhulaha | u kavhiwa | u thivhela u kavhiwa | Zwitshili | Zwikho khonono zwitukusa zwi no tshila | Ndi zwitshila zwo kundaho mushonga wa u zwinvhulaha |
| --- | --- | --- | --- | --- | --- | --- | --- | --- |
|  | mushonga wa fhugudza nyaluwo ya zwitshili | U kona ha zwitshiliulwa na mushonga wa u zwi vhulaha | Ndi ndila ya ufunza vhafhi vha mushonga wa u zwi vhulaha | u kavhiwa | Zwithivhela u kavhiwa | Zwitshili | Zwikho khonono zwitukusa zwi no tshila | Ndi zwitshila zwo kundaho mushonga wa u zwinvhulaha |
| **IsiNdebele** | Mshoga | ukungabheregi kwe shlahla |  | Indruga | Isivhikhela bolwlao | ndonga | Bolwelwe |  |
|  | kwemithi elwa nama gciwane | ukumelana namagciwane | ubuphathi bama antibiotic |  | Ukuvikela ukutheleleka | amagciwane | Silwanyana |  |
| **Xitsonga** | Vulawuri byavuvobyi | Nkeyivelo wa vutshunguri | Vatirwi va vutsunguri | Ntluketo wa vuvabyi | Nsivela ntluleto | switsongwa tsongwani | Nxopelo wa switsongwatsonwani |  |
|  |  |  |  | Ku ngheniwa | Ku sivela | Xitsongatsongani |  |  |
|  | Muri |  |  |  |  |  |  |  |
| **Setswana** | Selwantsa malwetsi | Segana golwantsa malwetsi | Morutiwa wa selwantsa malwetsi | Infectioni | Thibelano ya infectioni | kokwana thloko |  | Dinta tse kgolo |
|  | Ntwantsha malwetsi | Hlolego yago lwantsa malwetsi |  | Kokwanahloko | thibelano ya infectioni | mohuta wa dikokwanahloko | Kokwana | Ke leuba |
|  | Melemo e e bolayang merage |  |  | Tshwaetso | Thibela tshwaetsego | Twatsi |  | Twatsi e sa alafegeng |
|  |  |  |  | Mogare |  |  |  |  |
|  | Selwantshi sa malwetsi | Go gana kedi antibiotic |  | Go tshwaitsega | Se thibela malwetsi | Lesole la go baka bolwetsi |  |  |
|  | Molemo o bolayang mogare | Kganetso ya mareolemo o obolayang mo |  | Tshoaelo | Thibela tsoaetso | Mogare |  |  |
|  |  |  |  | Fetelelo | Thibelo ya fetelelo |  |  |  |
| **Afrikaans** | Medisyne | Onfang | Persoon wat dit gebruik | moenie insteek nie | Moenie insteek virander mense |  | Mooi asem uithaal |  |
